# Cardiovascular Disease Risk in Children With Chronic Kidney Disease: Impact of Apolipoprotein C-II and Apolipoprotein C-III

**DOI:** 10.3389/fped.2021.706323

**Published:** 2021-08-12

**Authors:** Wei-Ling Chen, You-Lin Tain, Hung-En Chen, Chien-Ning Hsu

**Affiliations:** ^1^Division of Pediatric Nephrology, Kaohsiung Chang Gung Memorial Hospital and Chang Gung University College of Medicine, Kaohsiung, Taiwan; ^2^Department of Pharmacy, Kaohsiung Chang Gung Memorial Hospital, Kaohsiung, Taiwan; ^3^School of Pharmacy, Kaohsiung Medical University, Kaohsiung, Taiwan

**Keywords:** chronic kidney disease, ambulatory blood pressure monitoring, apolipoprotein C, cardiovascular disease, children, congenital anomalies of the kidney and urinary tract, hypertension, proteomics

## Abstract

Cardiovascular disease (CVD) is an evolving process that begins in the early stages of chronic kidney disease (CKD) in children. Several surrogate markers, such as ambulatory blood pressure monitoring (ABPM), left ventricular (LV) mass, and arterial stiffness assessment, allow for the early detection of subclinical CVD in pediatric CKD. Four groups of plasma samples (*n* = 3/group) from congenital anomalies of the kidney and urinary tract (CAKUT), as well as non-CAKUT patients with or without BP abnormalities, were studied to screen differentially expressed proteins using isobaric tags for relative and absolute protein quantification (iTRAQ)-based proteomics. As a result, 20 differentially expressed proteins associated with hypertension in children with CKD were discovered. Among them, apolipoprotein C-II (apoC-II) was found to have the highest abundance among the CKD patients with hypertension. As such, we hypothesized that apoC-II and apolipoprotein C-III (apoC-III) levels were related to BP abnormalities and CVD in children suffering from mild-to-moderate CKD. We examined their associations with surrogate markers of CV risk in 88 pediatric patients with CKD stages G1–G4. Children with CKD stages G2–G4 had a higher plasma apoC-II level than G1 patients (6.35 vs. 5.05 mg/dl, *p* < 0.05). We observed that ABPM abnormalities, LV mass, and arterial stiffness parameters were greater in CKD children who had stages G2–G4 than in those who had stage G1 (all *p* < 0.05). Plasma levels of apoC-II and apoC-III were positively correlated with total cholesterol, triglyceride, and low-density lipoprotein (LDL) (all *p* < 0.001). In multivariate linear regression analyses, apoC-II was correlated with a high LV mass index and an abnormal ABPM profile, and apoC-III was correlated with 24-h hypertension (*r* = 0.303, *p* = 0.003) and asleep hypertension (*r* = 0.379, *p* < 0.001). Early evaluations of apoC-II and apoC-III, ABPM, and surrogate markers of CV risk will aid in early preventative interventions to reduce the risk of CV in youths suffering from CKD.

## Introduction

Chronic kidney disease (CKD) is a challenge in global health. As well as being highly prevalent in adults (1), CKD has also been increasing in the pediatric population (2, 3). The causes of childhood CKD differ from those in adults, as the main causes of CKD in children are congenital anomalies of the kidney and urinary tract (CAKUT) (4). Cardiovascular disease (CVD) is the most common cause of morbidity and mortality in both adults and children with CKD (5). However, unlike in adults, overt features of CVD rarely exist in children (6).

Hypertension (HTN) is one of the most important risk factors for CVD. We and others have shown previously that more than 50% of children with early-stage CKD have blood pressure (BP) abnormalities in 24-h ambulatory BP monitoring (ABPM) (7, 8). In addition, several structural or functional surrogate markers have been used to assess CVD risk in pediatric CKD, such as ambulatory arterial stiffness index (AASI), pulse wave velocity (PWV), left ventricular (LV) mass, and LV mass index (LVMI) (9). CAKUT and HTN can both appear early on in life (10, 11). In light of this, identifying risk factors linking CAKUT to HTN is crucial for preventing the growing epidemic of diseases associated with CKD, as well as for gaining a greater understanding of CAKUT in the early stages of childhood CKD.

Currently, proteomics research has been extensively applied to identify proteins as biomarkers in various diseases, including kidney disease (12). In combination with mass spectrometry, isobaric tags for relative and absolute protein quantification (iTRAQ) has been useful in determining potential protein biomarkers (13). In the current study, we first used iTRAQ to conduct comparative proteome profiling of the plasma samples between CAKUT and non-CAKUT children with or without BP abnormalities, and we did so to identify disease-specific proteins. The iTRAQ-based proteomic method identified potential biomarkers, apolipoprotein C-II (apoC-II) and apolipoprotein C-III (apoC-III), which could be validated in our subsequent studies to predict CV risk in children with CKD.

ApoC-II and apoC-III belong to the apolipoprotein multigene family. Apolipoproteins are proteins that bind to lipids to form lipoproteins whose function is to transport lipids for metabolism and utilization. Plasma apoC-II can activate lipoprotein lipase (LPL) and thus reduces plasma triglyceride levels (14). Elevated apoC-III levels are associated with increased CVD risk, as they are an important mediator of atherogenic dyslipidemia (14). Since apoC-III is mainly excreted by the kidney (15), increased plasma levels of apoC-III and triglyceride were reported in patients with moderate CKD due to delayed apoC-III catabolism (16). Lipid abnormalities are common in children with CKD, especially in the advanced stage of the disease (17). However, there are still very limited data on the earlier stages of childhood CKD. Although dyslipidemia is a risk factor for CVD in patients with CKD, no information currently exists regarding the association between apoC-II/apoC-III and CV risk in the early stages of childhood CKD. Here, we hypothesized that apoC-II and apoC-III levels were related to BP abnormalities and CVD in children suffering from mild-to-moderate CKD. As such, we aimed to estimate the associations between plasma levels of apoC-II and apoC-III, BP abnormalities, and CVD risk markers in children with mild-to-moderate CKD.

## Materials and Methods

### Patients and Study Design

The study protocol complied with the ethical guidelines of the 1964 Declaration of Helsinki and its later amendments. Before this observational cohort commenced, appropriate approval was obtained from the Institution Review Board and Ethics Committee of Chang Gung Medical Foundation, Taoyuan, Taiwan (permit numbers: 201601181A3 and 201701735A3C501). We used the KDIGO guideline to define the stages of CKD (18). The estimated glomerular filtration rate (eGFR) was calculated according to the Schwartz formula (19). CKD children with eGFR ≥90 ml/min/1.73 m^2^ were categorized as stage G1; G2 denotes eGFR 60–89 ml/min/1.73 m^2^; G3 represents 30–59 ml/min/1.73 m^2^; and G4 stands for 15–29 ml/min/1.73 m^2^. Inclusion criteria for CKD subjects were the following: children and adolescents aged 3–18 years with CKD stages G1–G4. CKD patients who were pregnant, already at eGFR <15 ml/min/1.73 m^2^, or on dialysis, as well as those who had congenital heart disease or renal transplant, or were unable to receive CV assessment, were excluded from the study. All of the participants provided signed informed consent for study. The causes of CKD were divided into two categories, CAKUT or non-CAKUT, as described previously (20). CAKUT structural anomalies included renal agenesis, posterior urethral valves, duplex collecting system, multi-cystic kidney dysplasia, kidney dysplasia, kidney hypoplasia, horseshoe kidney, and ureter abnormalities (4).

This ancillary study was performed in a subgroup of 88 participants who received the analysis of plasma apoC-II and apoC-III levels and CV assessments simultaneously. Fasting blood samples were drawn and aliquoted, and spot urine samples were collected and stored in a −80°C freezer until analysis. Blood urea nitrogen (BUN), creatinine (Cr), uric acid, glucose, hemoglobin, hematocrit, sodium, potassium, calcium, phosphate, urine total protein-to-Cr ratio, total cholesterol, triglyceride, and low-density lipoprotein (LDL) were measured by the hospital central laboratory, as described previously (8). Plasma apoC-II concentrations were determined by an ELISA kit (Catalog No. EHAPOC2; Invitrogen, Thermo Fisher Scientific Inc., Carlsbad, CA, USA), and inter-assay coefficients of variations were <12%. Plasma apoC-III concentrations were analyzed by an ELISA kit (Catalog No. EHAPOC3; Invitrogen, Thermo Fisher Scientific Inc., Carlsbad, CA, USA), and inter-assay CVs were <12%.

### Isobaric Tags for Relative and Absolute Protein Quantification Labeling and Liquid Chromatography–Tandem Mass Spectrometry Analysis

Four groups of plasma samples (*n* = 3/group) from CAKUT and non-CAKUT patients, either with or without BP abnormalities, were studied using iTRAQ labeling and liquid chromatography–tandem mass spectrometry (LC-MS/MS). For iTRAQ labeling proteomic technology, pooling small-size samples into groups and evaluating pools rather than individuals are commonly applied to identify potential differential expressed biomarkers between groups (21–23). This ancillary study was performed in the subgroups of 12 and 88 participants, which were recruited from the same cohort study with power calculation to determine the effective sample size. Abnormal BP profile was defined by ABPM. ITRAQ labeling was performed according to previously reported methods (21). In brief, the plasma samples were subjected to high-abundance protein depletion with the Pierce Top 12 Abundant Protein Depletion Spin Columns (Thermo Fisher Scientific Inc., Waltham, MA, USA). Later, the plasma samples of three subjects were evenly pooled, resulting in four samples: CAKUT+HTN, CAKUT+N, non-CAKUT+HTN, and non-CAKUT+N. The four pooled plasma samples were then subjected to sample preparation with the iTRAQ Reagents Multiplex Kit (AB Sciex, Redwood City, CA, USA). After passing the standard QC check, the labeled samples were analyzed with LC/Q-Exactive Orbitrap MS (Thermo Fisher Scientific Inc., Waltham, MA, USA) for 24 h. The raw data were analyzed with Proteome Discoverer v2.4 (Thermo Fisher Scientific Inc., Waltham, MA, USA). Database search was performed using the MASCOT 2.5 database (Matrix Science Inc., Boston, MA, USA) for protein identification. As a result, the relative abundances of detected proteins were obtained. Proteins were considered to be differentially expressed if the difference was statistically significant (*p* < 0.05) and the fold change was >1.5 or <0.67.

### Blood Pressure Measurement and Sonography

Subjects were instructed to measure office BP in the usual clinical fashion. Children rested for 5 min prior to measurement. Three seated, non-dominant arm BP measurements were recorded at 1-min intervals. The ABPM was conducted using an Oscar II monitoring device (SunTech Medical, Morrisville, NC, USA) over a 24-h period for subjects aged 6–18 years. An experienced specialist nurse performed in-office test measurements to ensure comfort and adequate fitting (8). The device was programmed to measure BP at 20-min intervals from 7 a.m. to 10 p.m. and at 30-min intervals from 10 p.m. to 7 a.m. Waking and sleep periods were defined by self-report diaries, and activities that might influence BP measurements were recorded. An abnormal ABPM profile was determined by (1) daytime BP, nighttime BP, systolic BP (SBP), or diastolic BP (DBP) ≥95th percentile according to ABPM reference data (24); (2) daytime, nighttime, systolic, or DBP load ≥25%; and (3) a sleep decrease in BP load by <10% compared with average awake BP load. The AASI, an indirect arterial stiffness index, was calculated from one minus the slope of the DBP on SBP during ABPM (18). Echocardiographic examination was carried out by pediatric cardiologists using a Philips IE33 system machine (Philips, Bothell, WA, USA). The parasternal long-axis or short-axis views of the left ventricle were obtained by M-mode echocardiography to calculate LV mass. The LVMI was calculated by indexing LV mass to height^3^ (25). PWV, an index of arterial stiffness, was determined by echo-tracking methods using the ProSound α7 ultrasound (ProSound α7, e-TRACKING system; Aloka Co., Tokyo, Japan) (8).

### Statistical Analysis

Continuous variables are presented as medians and inter-quartile ranges (IQRs; 25th−75th percentile), while categorical variables are described as numbers and percentages. The Mann–Whitney U-test or chi-square test was used to test the differences in variables between two groups. Spearman's rank correlation coefficient was used to determine the associations between variables. A linear regression model was performed, followed by stepwise multivariable analyses for integrating relevant parameters to explain apoC-II, apoC-III, and CV risk markers. A *p* < 0.05 was considered statistically significant. All analyses were performed using Statistical Package for the Social Sciences (SPSS) software 14.0 (Chicago, IL, USA).

## Results

### Differentially Expressed Proteins Identified by Proteomic Analysis

The plasma samples from patients with different causes of CKD (CAKUT vs. non-CAKUT) and patients with or without abnormal ABPM profiles were studied using iTRAQ labeling and LC-MS/MS. As a result, 502 proteins were identified with a <1% false discovery rate (FDR) and a minimum of two peptides (the overall iTRAQ data are available in Supplementary Table 1). Next, we analyzed the quantitative differences between the groups of patients. As shown in Table 1, the first set (HTN/N) of differentially expressed proteins consisted of those who had BP abnormalities, regardless of whether their underlying CKD was CAKUT or non-CAKUT, including apoC-II, platelet factor 4, and calmodulin, etc. A total of 20 proteins were identified: one-half was upregulated, and the other was downregulated. The second set (CAKUT-HTN/N) of differentially expressed proteins consisted only of those who differed significantly between CAKUT patients with abnormal and normal BPs. The third set of altered proteins comprised those who differed significantly between abnormal and normal BP in CVD patients with non-CAKUT. The abundance of apoC-II was 1.71-, 1.78-, and 1.65-fold higher in three different sets of patients (Table 1). Although it did not reach significance when comparing BP abnormalities in CAKUT patients, alterations in the abundance of apo-C-III between in the first (HTN/N) and third sets (non-CAKUT-HTN/N) were evident (Table 1). Among the proteins detected by proteomics, we selected apoC-II and apoC-III for subsequent studies.

**Table 1:** Comparison of the abundance of proteins differentially expressed in children with CKD and blood pressure abnormalities.

**Protein name**	**Protein abundance**				**Fold change**		
	**CAKUT** **+HTN (a)**	**CAKUT** **+ N (b)**	**Non-CAKUT** **+ HTN (c)**	**Non-CAKUT** **+ N (d)**	**HTN/N** **(a + c)/(b + d)**	**CAKUT–HTN/N** **a/b**	**Non-CAKUT–HTN/N** **c/d**
Apolipoprotein C-II	115.56	64.80	124.00	75.00	1.71	1.78	1.65
Platelet factor 4	52.00	77.50	189.62	65.00	1.70	0.67	2.92
Calmodulin-1	78.31	79.30	158.19	66.60	1.62	0.99	2.38
Ras suppressor protein 1	71.56	77.80	164.95	68.40	1.62	0.92	2.41
14-3-3 protein zeta/delta	75.29	75.00	158.48	73.90	1.57	1.00	2.14
Transgelin-2	77.07	80.70	156.48	68.30	1.57	0.95	2.29
Peptidyl-prolyl *cis*-*trans* isomerase A	72.09	75.20	161.33	74.40	1.56	0.96	2.17
LIM and senescent cell antigen-like-containing domain protein 1	76.80	81.80	155.52	68.40	1.55	0.94	2.27
Apolipoprotein C-III	110.93	74.30	119.43	75.50	1.54	1.49	1.58
Vimentin	80.53	77.70	150.00	74.20	1.52	1.04	2.02
Pregnancy zone protein	59.11	161.80	94.95	72.10	0.66	0.37	1.32
Keratin, type II cytoskeletal 5	81.96	114.90	69.62	119.80	0.65	0.71	0.58
Keratin, type II cytoskeletal 1	86.76	123.00	58.48	117.90	0.60	0.71	0.50
Keratin, type II cytoskeletal 6	60.09	122.50	84.95	120.60	0.60	0.49	0.70
Keratin, type II cytoskeletal 9	85.16	132.80	58.86	109.60	0.59	0.64	0.54
Keratin, type II cytoskeletal 2	77.24	117.60	65.90	126.30	0.59	0.66	0.52
Keratin, type II cytoskeletal 10	81.69	107.30	60.76	137.00	0.58	0.76	0.44
Serum amyloid A-1 protein	52.71	63.80	59.05	214.80	0.40	0.83	0.27
Serum amyloid A-2 protein	54.13	66.80	56.10	213.40	0.39	0.81	0.26
C-reactive protein	41.60	70.40	51.62	228.60	0.31	0.59	0.23

*Difference identified as significant (fold change >1.5 or <0.67). Data tabulated according to HTN/N and ordered from high to low*.

*CAKUT, congenital anomalies of the kidneys and urinary tract; HTN, hypertension; N, normal blood pressure*.

### Cohort Characteristics

The characteristics of the study participants are summarized in Table 2. In total, there were 88 children and adolescents with CKD stages G1–G4, including 56 G1 subjects (63.6%), 23 G2 subjects (26.1%), seven G3 subjects (8%), and two G4 subjects (2.3%). The majority of the participants (96.6%) recruited for the present study fell into the mild-to-moderate categories. Our study population had a median age of 13.6 years; 42% of our participants were male and 55.7% had CAKUT. Nearly 36% (32/88) of the CKD children and adolescents were diagnosed with HTN according to their office BP readings. CKD children and adolescents were divided into two groups based on two levels of renal function. The G1 group had an eGFR ≥90ml/min/1.73 m^2^, and the G2–G4 group had an eGFR <90 ml/min/1.73 m^2^. CKD G2–G4 patients were older, were predominantly female, and had higher systolic and diastolic office BPs, BUN, Cr, and uric acid; however, they also had lower eGFR and phosphate than had G1 patients.

**Table 2:** Clinical anthropometric and biomedical characteristics of study population (*n* = 88).

**CKD stage**	**G1**	**G2–G4**	***p*-Values**
Characteristics	*N* = 56	*N* = 32	
Age, years	12.3 (9.5–14.5)	15.8 (11.5–17.7)	0.002^*^
Male	30 (53.6%)	7 (21.9%)	0.003^*^
CAKUT	27 (48.2%)	22 (68.8%)	0.049^*^
Body height, percentile	50 (25–85)	25 (15–69)	0.329
Body weight, percentile	50 (17.5–85)	50 (15–85)	0.899
Body mass index, kg/m^2^	18.9 (16.8–22.5)	19.9 (17–24.7)	0.183
Systolic blood pressure, mmHg	109 (101–120)	124 (112–134)	0.002^*^
Diastolic blood pressure, mmHg	70 (64–77)	75 (68–84)	0.036^*^
Hypertension (by office BP)	15 (26.8%)	17 (53.1%)	0.013^*^
Blood urea nitrogen, mg/dl	11.5 (10–14)	14.5 (12–19.5)	<0.001^*^
Creatinine, mg/dl	0.55 (0.5–0.63)	0.93 (0.76–1.21)	<0.001^*^
Estimated glomerular filtration rate, ml/min/1.73 m^2^	110 (100–119)	73 (56–82)	<0.001^*^
Urine total protein-to-creatinine ratio, mg/g	68.5 (30.3–354.6)	116.7 (24.7–971.7)	0.708
Hemoglobin, g/dl	13.5 (12.8–14.3)	13.8 (12.5–15.3)	0.316
Hematocrit, %	39.9 (38.1–41.8)	41.1 (37.5–45.1)	0.410
Fasting glucose, mg/dl	90 (86–94.8)	88 (85.3–93)	0.667
Uric acid, mg/dl	4.9 (3.9–6)	7.2 (6.1–7.7)	<0.001^*^
Sodium, mEq/L	140 (139–142)	141 (139–142)	0.755
Potassium, mEq/L	4.3 (4.2–4.5)	4.4 (4.2–4.6)	0.148
Calcium, mg/dl	9.7 (9.5–9.9)	9.8 (9.5–9.9)	0.741
Phosphate, mg/dl	4.7 (4.3–5.2)	4.3 (3.9–4.8)	0.024^*^

*Data given as medians (IQR) or n (%)*.

*CAKUT, congenital anomalies of the kidneys and urinary tract*.

* *p < 0.05 by the Mann–Whitney U-test*.

### Lipids and Apolipoprotein C

As presented in Table 3, plasma apoC-II levels were higher in children with CKD G2–G4 than G1 patients. In the current study, there was no difference in apoC-III levels between the two groups. The results obtained from the present study demonstrated that lipid abnormalities were not common in children with early-stage CKD, with 13.4% (12/88) having LDL levels of >130 mg/dl, 18.2% (16/88) with total cholesterol levels of >200 mg/dl, and 14.8% (13/88) with triglyceride levels of >130 mg/dl. Although lipid abnormalities are associated with lower GFR and are common in children with advanced CKD (17), we observed no difference between the G2–G4 and G1 groups in our lipid profile findings. None of the participants received lipid-lowering medication.

**Table 3:** Plasma levels of apolipoprotein C-II, C-III, and lipids of study population (*n* = 88).

**CKD stage**	**G1**	**G2–G4**
	*N* = 56	*N* = 32
Apolipoprotein C-II, mg/dl	5.05 (3.87–6.44)	6.35 (4.72–8.93)^*^
Apolipoprotein C-III, mg/dl	8.46 (7.02–10.01)	8.89 (7.38–11.67)
Total cholesterol, mg/dl	164 (140–184)	156 (143–197)
Low-density lipoprotein, mg/dl	83.5 (65.3–108.5)	92.3 (70.3–125.3)
Triglyceride, mg/dl	69.5 (51.3–90.8)	85 (59.5–119.8)

*Data given as medians (IQR)*.

*CKD, chronic kidney disease; IQR, inter-quartile range*.

* *p < 0.05 by the Mann–Whitney U-test*.

**Figure 1:**
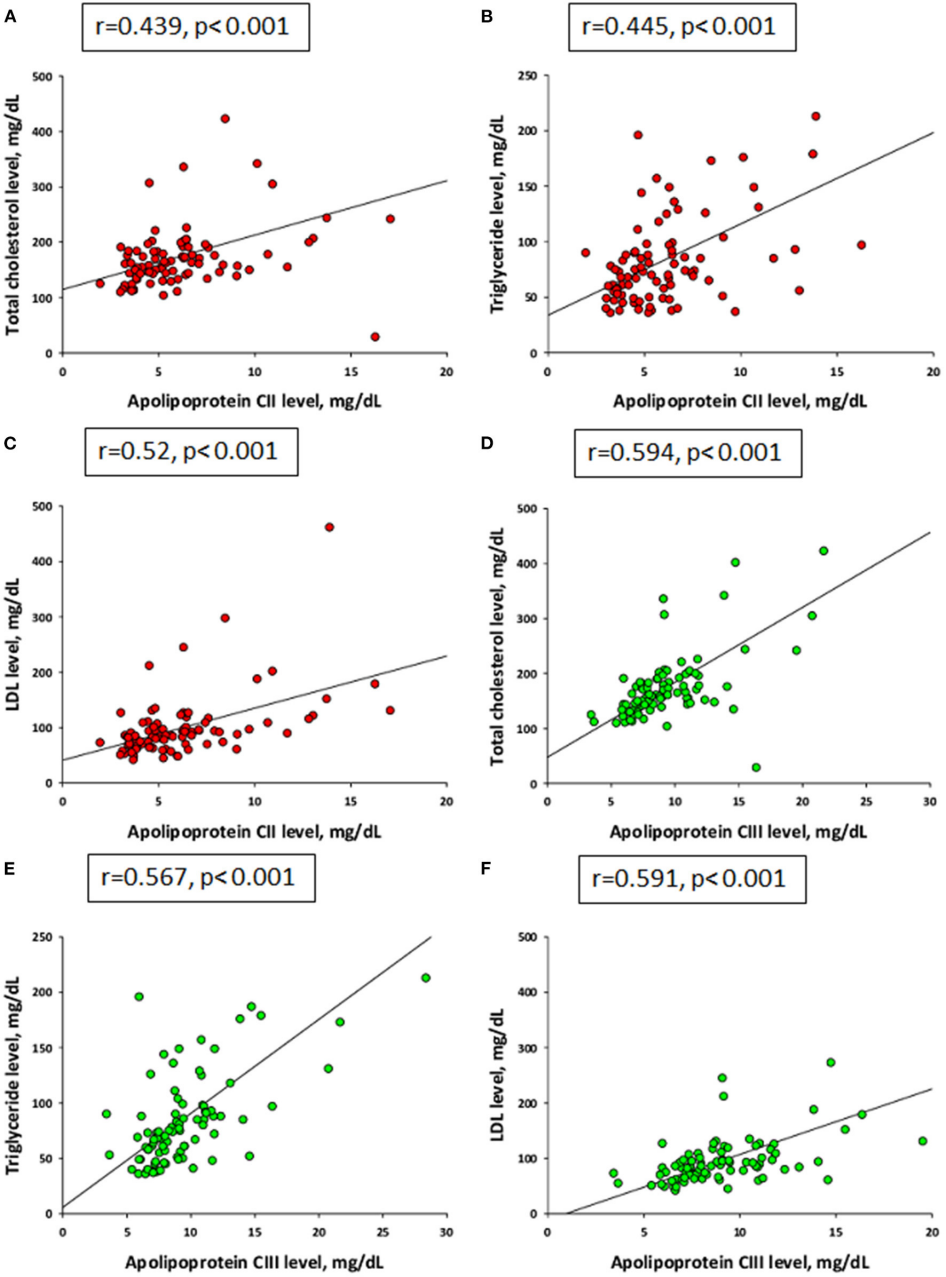
Correlations of plasma levels between apolipoprotein C-II (red) and **(A)** total cholesterol, **(B)** triglyceride, and **(C)** low-density lipoprotein (LDL). Plasma levels between apolipoprotein C-III (green) and **(D)** total cholesterol, **(E)** triglyceride, and **(F)** LDL by Spearman's correlation coefficient.

Analyses that evaluated the existence of associations between plasma apoC-II and apoC-III levels and lipids, as evaluated by data pooled from all subjects, indicated that there were strong positive correlations between the plasma apoC-II level and total cholesterol (Figure 1A, *r* = 0.439, *p* < 0.001), triglyceride (Figure 1B, *r* = 0.445, *p* < 0.001), and LDL (Figure 1C, *r* = 0.52, *p* < 0.001). Additionally, apoC-III was positively correlated with total cholesterol (Figure 1D, *r* = 0.594, *p* < 0.001), triglyceride (Figure 1E, *r* = 0.567, *p* < 0.001), and LDL (Figure 1F, *r* = 0.591, *p* < 0.001). The association between plasma eGFR and apoC-II and apoC-III levels was calculated, and no significant correlation was found between eGFR, apoC-II (*r* = −0.236, *p* = 0.027), and apoC-III (*r* = −0.09, *p* = 0.402).

### Cardiovascular Assessment and Ambulatory Blood Pressure Monitoring

PWV and AASI are both arterial stiffness parameters used for CV risk assessment (9, 26). Our data showed that PWV and AASI were greater in patients with CKD stages G2–G4 than in G1 patients. LV mass was also higher in the G2–G4 group, while LVMI did not differ between the two groups (Table 4).

**Table 4:** Cardiovascular assessments of study population (*n* = 88).

**CKD stage**	**G1**	**G2–G4**
	*N* = 56	*N* = 32
PWV	3.8 (3.4–4.3)	4.1 (3.8–4.7)^*^
AASI	0.35 (0.23–0.46)	0.42 (0.33–0.54)^*^
Left ventricular mass (g)	87.9 (65.5–109)	112.5 (84.5–156.5)^*^
LVMI (g/m^2.7^)	29.1 (25.8–34.1)	31.5 (27.5–36.8)

*Data are medians (IQR)*.

*PWV, pulse wave velocity; AASI, ambulatory arterial stiffness index; LVMI, left ventricular mass index; CKD, chronic kidney disease*.

* *p < 0.05 by the Mann–Whitney U-test*.

The frequency of HTN was higher on the basis of ABPM compared with office BP. Overall, 56.8% (50/88) of participants had at least one BP load abnormality on ABPM, including 12 subjects (13.6%) with 24-h BP or nighttime BP >95th percentile, 14 subjects (15.9%) with daytime BP >95th percentile, 32 subjects (36.4%) with a BP load ≥95th percentile, and 36 patients (40.9%) with a non-dipping nocturnal BP profile (Table 5).

**Table 5:** Plasma levels of apolipoprotein C-II and C-III vs. ABPM profile in CAKUT and non-CAKUT CKD children and adolescents.

	**CAKUT (** ***n*** **= 49)**			**Non-CAKUT (** ***n*** **= 39)**		
		**ApoC-II**	**ApoC-III**		**ApoC-II**	**ApoC-III**
**ABPM profile**	***N***	**Median (25th−75th)**	**Median (25th−75th)**	***N***	**Median (25th−75th)**	**Median (25th−75th)**
**24-h BP**						
Abnormal	4	4.68 (3.86–6.08)	7.15 (6.14-8.38)	8	7.35 (5.04–13.86)	13.15 (8.5–20.33)
Normal	45	5.36 (4.00–6.73)	8.58 (7.02-10.94)	31	4.82 (4.03–7.11)	9.03 (7.49–10.83)
**Awake BP**						
Abnormal	5	4.69 (4.13–6.47)	7.37 (6.32–8.13)	9	6.25 (3.93–13.82)	10.82 (6.93–19.01)
Normal	44	5.32 (3.93–6.73)	8.67 (7.01–10.95)	30	5.09 (4.14–7.37)	9.06 (7.74–10.9)
**Asleep BP**						
Abnormal	4	4.24 (3.65–4.69)	7.15 (6.14–12.84)	8	11.1 (5.79–15.67)^*^	15.11 (10.44–20.33)^*^
Normal	45	5.63 (4.29–6.73)	8.58 (7.02–10.8)	31	4.82 (4.03–7.08)^*^	8.87 (7.49–10.5)^*^
**BP load**						
Abnormal	16	4.68 (3.61–6.17)^*^	7.39 (6.53–8.72)	16	5.94 (3.55–13.56)	10.42 (7.52–15.29)
Normal	33	5.63 (4.47–7.55)^*^	8.94 (7.14–10.99)	23	5.36 (4.44–7.11)	9.03 (7.82–10.83)
**Night dipping**						
Abnormal	22	4.57 (3.65–6.34)^*^	7.69 (6.6–9.2)	14	5.99 (3.76–9.01)	9.99 (7.47–12.58)
Normal	27	6.23 (5.11–7.42)^*^	8.75 (7.25–11.03)	25	5.36 (4.31–8.25)	9.03 (7.86–12.99)
**ABPM profile**						
Abnormal	28	4.81 (3.83–6.29)^*^	7.83 (6.67–9.41)	22	6.3 (3.76–11.27)	9.9 (7.47–12.58)
Normal	21	6.44 (5.19–7.55)^*^	8.98 (7.04–11.32)	17	4.82 (4.31–7.22)	8.87 (7.87–9.99)

*ABPM, ambulatory blood pressure monitoring; CAKUT, congenital anomalies of the kidney and urinary tract; CKD, chronic kidney disease; BP, blood pressure*.

* *p < 0.05 abnormal vs. normal group by the Mann–Whitney U-test*.

### Cardiovascular Risk Markers Associated With Apolipoprotein C-II and Apolipoprotein C-III

Analysis of plasma apoC-II and apoC-III levels, stratified according to the ABPM profile, showed that there was a difference between the CAKUT and non-CAKUT groups. In Table 5, we compared the difference of apoC-II and apoC-III levels between abnormal and normal ABPM profiles in CAKUT and non-CAKUT patients, separately. It shows that plasma apoC-II level was significantly lower in CAKUT patients with a high BP load, non-night dipping, and an abnormal ABPM profile. Nevertheless, analysis of apoC-III levels in different elements on ABPM profiles did not reveal any significant difference between CKD children with abnormal and normal profiles. However, in patients with non-CAKUT, plasma apoC-II and apoC-III levels were significantly higher in CKD children with abnormal asleep BP than in normal patients. Additionally, in Table 6, we compared these levels of apoC-II and apoC-III between the CAKUT and non-CAKUT patient groups, and we found that apoC-III levels were higher in non-CAKUT patients with abnormal 24-h BP, non-night dipping, and an abnormal ABPM profile than in those with CAKUT.

**Table 6:** Comparison of plasma apoC-II and apoC-III levels according to the ABPM profile between the CAKUT and non-CAKUT CKD children and adolescents.

		**ApoC-II**	**ApoC-III**
**ABPM profile**	***N***	**Median (25th−75th)**	**Median (25th−75th)**
**24-h BP**			
Abnormal			
CAKUT	4	4.68 (3.86-6.08)	7.15 (6.14–8.38)^*^
Non-CAKUT	8	7.35 (5.04-13.86)	13.15 (8.5–20.33)^*^
Normal			
CAKUT	45	5.36 (4.00-6.73)	8.58 (7.02–10.94)
Non-CAKUT	31	4.82 (4.03-7.11)	9.03 (7.49–10.83)
**Awake BP**			
Abnormal			
CAKUT	5	4.69 (4.13–6.47)	7.37 (6.32–8.13)
Non-CAKUT	9	6.25 (3.93–13.82)	10.82 (6.93–19.01)
Normal			
CAKUT	44	5.32 (3.93–6.73)	8.67 (7.01–10.95)
Non-CAKUT	30	5.09 (4.14–7.37)	9.06 (7.74–10.9)
**Asleep BP**			
Abnormal			
CAKUT	4	4.24 (3.65–4.69)^*^	7.15 (6.14–12.84)
Non-CAKUT	8	11.1 (5.79–15.67)^*^	15.11 (10.44–20.33)
Normal			
CAKUT	45	5.63 (4.29–6.73)	8.58 (7.02–10.8)
Non-CAKUT	31	4.82 (4.03–7.08)	8.87 (7.49–10.5)
**BP load**			
Abnormal			
CAKUT	16	4.68 (3.61–6.17)	7.39 (6.53–8.72)
Non-CAKUT	16	5.94 (3.55–13.56)	10.42 (7.52–15.29)
Normal			
CAKUT	33	5.63 (4.47–7.55)	8.94 (7.14–10.99)
Non-CAKUT	23	5.36 (4.44–7.11)	9.03 (7.82–10.83)
**Night dipping**			
Abnormal			
CAKUT	22	4.57 (3.65–6.34)	7.69 (6.6–9.2)^*^
Non-CAKUT	14	5.99 (3.76–9.01)	9.99 (7.47–12.58)^*^
Normal			
CAKUT	27	6.23 (5.11–7.42)	8.75 (7.25–11.03)
Non-CAKUT	25	5.36 (4.31–8.25)	9.03 (7.86–12.99)
**ABPM profile**			
Abnormal			
CAKUT	28	4.81 (3.83–6.29)	7.83 (6.67–9.41)^*^
Non-CAKUT	22	6.3 (3.76–11.27)	9.9 (7.47–12.58)^*^
Normal			
CAKUT	21	6.44 (5.19–7.55)	8.98 (7.04–11.32)
Non-CAKUT	17	4.82 (4.31–7.22)	8.87 (7.87–9.99)

*ABPM, ambulatory blood pressure monitoring; CAKUT, congenital anomalies of the kidney and urinary tract; CKD, chronic kidney disease; BP, blood pressure*.

* *p < 0.05 CAKUT vs. non-CAKUT group by the Mann–Whitney U-test*.

Then, we performed multivariate linear regression analyses to specify the exact role of apoC-II and apoC-III in individual CV risk (Table 7). A multivariate linear regression model using the stepwise selection was applied for sex, age, CAKUT, and eGFR. In the best predictive model (*r* = 0.543, *p* < 0.001), plasma apoC-II level positively correlated with 24-h SBP (*p* = 0.004). Additionally, an association between 24-h DBP (*p* = 0.037), awake SBP (*p* = 0.006), asleep SBP (*p* = 0.011), awake DBP (*p* = 0.021), DBP load (*p* = 0.011), and LVMI (*p* = 0.033) was found in the adjusted regression model when controlling for age. ApoC-III level showed a positive correlation with 24-h hypertension (*p* = 0.003), awake HTN (*p* = 0.039), and asleep HTN (*p* < 0.001). Although Tables 5, 6 did not adjust CKD stage, Table 7 reveals apoC-II and apoC-III to have a positive correlation with CV risk markers in children with mild-to-moderate CKD, regardless of eGFR. These findings suggest a strong influence of apoC-II and apoC-III on surrogate markers of CV risk.

**Table 7:** Adjusted regression model estimates of the association between plasma apoC-II, apoC-III, and surrogate markers of CV risk.

**Dependent variable**	**Explanatory variable**	**Adjusted^a^**		**Model**	
		***Beta***	***p*-Value**	**r**	***p*-Value**
24-h SBP	ApoC-II	0.269	0.004	0.543	<0.001
24-h DBP	ApoC-II	0.209	0.037	0.423	<0.001
Awake SBP	ApoC-II	0.27	0.006	0.479	<0.001
Asleep SBP	ApoC-II	0.253	0.011	0.437	<0.001
Awake DBP	ApoC-II	0.233	0.021	0.407	<0.001
Diastolic BP load	ApoC-II	0.261	0.011	0.391	0.001
LVMI	ApoC-II	0.218	0.033	0.375	0.002
24-h hypertension	ApoC-III	0.303	0.003	0.425	<0.001
Awake hypertension	ApoC-III	0.209	0.039	0.421	0.001
Asleep hypertension	ApoC-III	0.379	<0.001	0.379	<0.001

*LVMI, left ventricular mass index; apoC-II, apolipoprotein C-II; apoC-III, apolipoprotein C-III; CV, cardiovascular; SBP, systolic blood pressure; DBP, diastolic blood pressure; eGFR, estimated glomerular filtration rate; CAKUT, congenital anomalies of the kidney and urinary tract*.

a *Adjusted for age, sex, eGFR, and CAKUT*.

## Discussion

**Figure 2:**
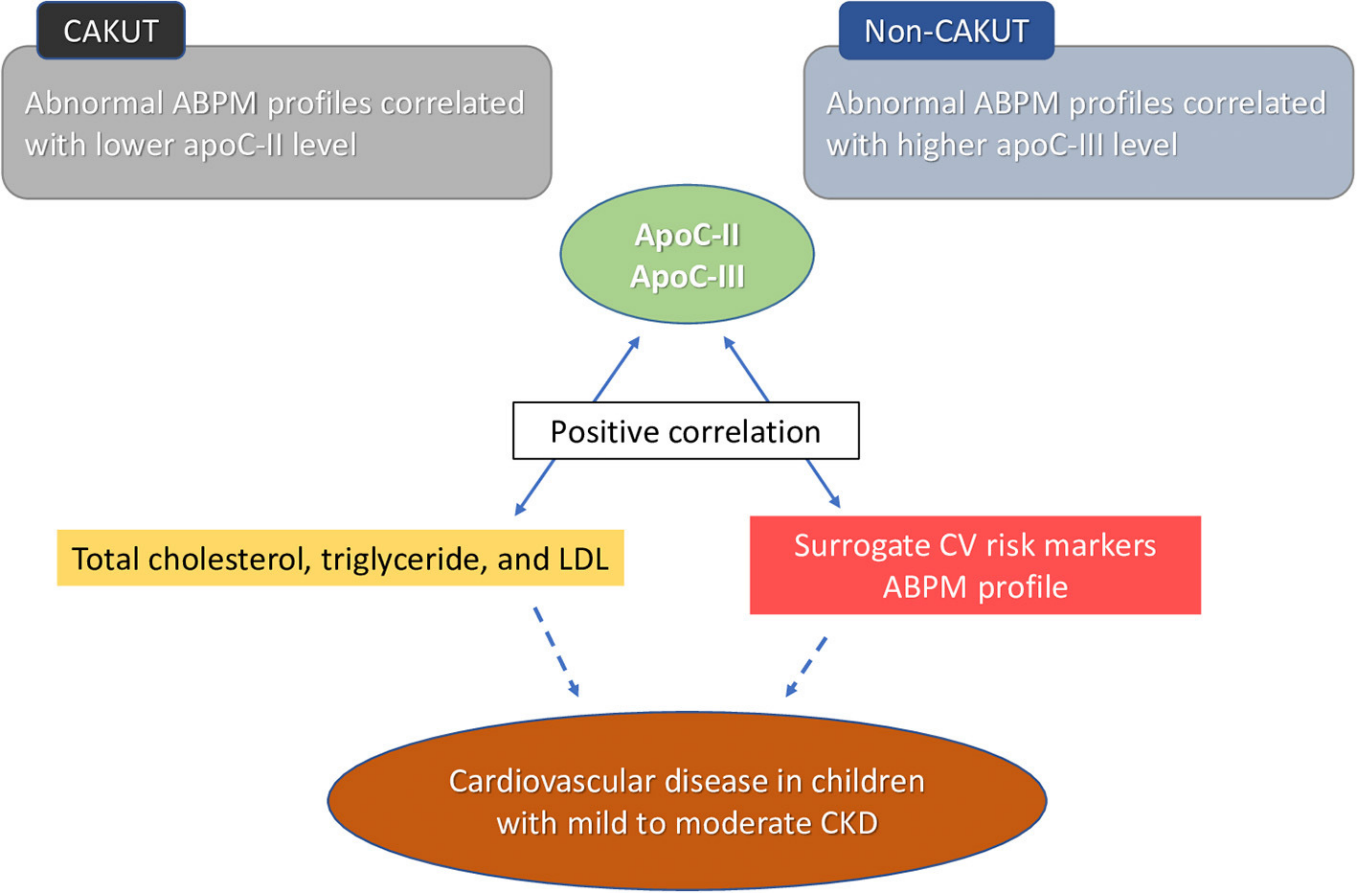
Plasma apolipoprotein C-II and C-III levels have a positive correlation with lipid profile, including total cholesterol, triglyceride, and low-density lipoprotein (LDL). Plasma apolipoprotein C-II and C-III levels have a positive correlation with surrogate cardiovascular (CV) risk markers. Abnormal ambulatory blood pressure monitoring (ABPM) profile was associated with lower apoC-II level in congenital anomalies of the kidney and urinary tract (CAKUT) patient and higher apoC-III levels in non-CAKUT patients. These correlations suppose a negative impact of apolipoprotein C-II and C-III on cardiovascular risk in the early stages of childhood chronic kidney disease (CKD).

This study provides better insight into the negative impacts of apoC-II and apoC-III associated with cardiovascular (CV) risk in the early stages of childhood CKD. Our significant findings are as follows: (1) using iTRAQ-based proteomic analysis, we identified 20 differentially expressed proteins associated with HTN in children with CKD, including apoC-II and apoC-III; (2) children with CKD stages G2–G4 had a higher plasma apoC-II level compared with stage G1 patients; (3) apoC-II and apoC-III both positively correlated with total cholesterol, triglyceride, and LDL in the plasma; (4) plasma apoC-II level was lower in CAKUT patients with high BP load, non-night dipping, and abnormal ABPM profile, whereas apoC-III level was higher in non-CAKUT children with high asleep BP; and (5) apoC-II and apoC-III had a positive correlation with CV risk markers, such as LVMI and ABPM profile, in children with mild-to-moderate CKD (Figure 2).

To our knowledge, no studies have been conducted regarding the association between apoC-II, apoC-III, and CV risk markers in the early stages of childhood CKD. Our data are consistent with prior studies showing that certain surrogate markers of CV risk already exist in children with early stages of CKD, such as abnormal ABPM (8, 20, 27), high PWV (8), increased LV mass (7, 28), and elevated AASI (8, 29). As expected, the severity of CKD is associated with high PWV, AASI, and LV mass in the present study. Even in stage G1, up to 33% of youths with CKD had abnormal ABPM profiles. The high prevalence of BP abnormalities in our study supports the case for the early ABPM evaluation in youth with early-stage CKD, especially in those at high risk of CVD (30).

Using an iTRAQ MS/MS approach, we screened differentially expressed proteins between the groups of CKD children with or without ABPM abnormalities and found 10 upregulated proteins and 10 downregulated proteins. In agreement with a previous study using an iTRAQ-based proteomic approach to identify apolipoproteins and proteins involved in lipid metabolism related to CVD in adult CKD patients (31), our proteomic approach identified apoC-II and apoC-III and found that they might be linked to CVD risk markers in pediatric CKD.

A positive correlation has been observed between plasma apoC-II and triglyceride, and between apoC-III and triglyceride (32, 33). Our results go beyond prior research, showing that both apoC-II and apoC-III strongly correlated not only with triglyceride but also with total cholesterol and LDL in CKD children. Although dyslipidemia is considered a common comorbidity in children with CKD (34), it is typically more common in advanced CKD and severe in non-CAKUT. In the current study, only around 15% of children with early-stage CKD displayed lipid abnormalities. It is noteworthy that apoC-II was increased in children with CKD G2–G4 compared with G1 patients; this was not the case with lipid profiles. As lipoproteins were regulated by apolipoprotein, our findings suggest that apoC-II and apoC-III are closely related to CV risk markers in early-stage childhood CKD, which may precede impaired lipoprotein metabolism, as evaluated by alterations in lipid profiles.

It is still debated whether high plasma apoC-II is beneficial or harmful for CV health (14). Higher levels of apoC-II are associated with increased triglyceride-rich particles and alterations in high-density lipoprotein (HDL) particle distribution that could influence CVD risk (35). Table 5 demonstrates that an abnormal ABPM profile was correlated with low apoC-II in the CAKUT group, but it was also correlated with high apoC-II in the non-CAKUT group. Additionally, Table 7 reveals that plasma apoC-II level was positively correlated with several markers of BP abnormality and LVMI. LVMI is not only an index of target organ damage but also a risk factor for CVD in CKD patients (7). Our study is the first to show that there is a positive correlation between LVMI and plasma apoC-II levels in children with CKD. We previously observed that CAKUT was associated with a low occurrence of BP abnormalities and CKD progression (20). Prior studies reported that CKD patients with CAKUT survived longer than those without CAKUT due to a lower CV mortality (36, 37). Accordingly, it is possible that low apoC-II might be a compensatory mechanism to protect CAKUT children against BP abnormalities and subclinical CVD. As lipid-lowering medication such as statins and fibrates have been shown to reduce apoC-II concentrations (35), it seems important to understand whether these medications can affect CVD risk in children with CKD via the mediation of apoC-II. In the non-CAKUT group, high apoC-III seems to be a risk factor that is associated with subclinical CVD in CKD children as shown in Table 5. Therefore, whether apoC-II and apoC-III play differential roles in the development of CVD in CKD children with CAKUT vs. non-CAKUT children deserves further clarification.

Although increased plasma levels of apoC-III were reported in adult patients with CKD (16), our data showed that plasma levels were independent of severity in children with early-stage CKD (Table 3). Notably, in Table 6, we found that apoC-III levels were higher in non-CAKUT patients with abnormal 24-h BP, non-night dipping, and an abnormal ABPM profile than in those with CAKUT. This finding raises questions regarding whether apoC-III, rather than apoC-II, may be a substantial contributor to CV risk in non-CAKUT CKD children. As high levels of apoC-III represent a pro-atherogenic risk factor, and apoC-III-lowering agents reduce CV risk (16), the targeting of apoC-III metabolism may become a potential therapeutic approach in preventing CVD in CKD children. This point awaits further elucidation.

Our study was not without its limitations. First, we mainly studied apoC-II and apoC-III based on an iTRAQ-based proteomic analysis. The results of the present study raise a question regarding whether other apolipoproteins are also involved in the pathogenesis of CVD. The answer to this question should be determined. Importantly, the application of the proteomic techniques described herein also offers up other candidate proteins for future research. Second, we examined the differences between apoC-II, apoC-III, and surrogate markers of CV risk at two levels of renal function without recruiting normal controls. Although plasma levels of apoC-II and apoC-III determined in the current study are comparable with those published previously (14), whether their concentrations are different between children with and without CKD remains to be determined. Last, we are well aware that our samples of CKD children from one hospital may not be representative of the population as a whole. Besides, although pooling small size samples into groups are commonly applied in iTRAQ labeling proteomic technology, whether the small sample size can be representative of the other patients in any way needs further clarification. Future large-sample multicenter studies may be required to detect a true relationship.

## Conclusions

In the current study, the high prevalence of BP abnormalities and associated CV risk markers highlights the need to screen for CV risks in CKD children, even in the early stages. The iTRAQ-based proteomic analysis performed in this study identifies apoC-II and apoC-III that are related to BP abnormalities, showing that both are associated with several surrogate markers of CV risk in a pediatric CKD population. Although the mechanism underlying CVD attributed to apoC-II and apoC-III has not yet been fully elucidated, our results shed new light on the links between apoC-II, apoC-III, lipoproteins, and CV risk markers in children with mild-to-moderate CKD. A better understanding of the impact of apoC-II and apoC-III on CVD and CKD would aid in initiating treatment at an early stage with respect to improving CV outcomes in at-risk youths suffering from CKD.

## Data Availability Statement

The original contributions presented in the study are included in the article/Supplementary Material, further inquiries can be directed to the corresponding author.

## Ethics Statement

The studies involving human participants were reviewed and approved by Institution Review Board and Ethics Committee of Chang Gung Medical Foundation, Taoyuan, Taiwan (Permit numbers: 201601181A3 & 201701735A3C501). Written informed consent to participate in this study was provided by the participants' legal guardian/next of kin.

## Author Contributions

W-LC contributed to concept generation, methodology, data interpretation, drafting of the manuscript, critical revision of the manuscript, and approval of the article. Y-LT contributed to concept generation, methodology drafting of the manuscript, critical revision of the manuscript, and approval of the article. H-EC contributed to data interpretation, drafting of the manuscript, critical revision of the manuscript, and approval of the article. C-NH contributed to concept generation, data interpretation, methodology, drafting of the manuscript, critical revision of the manuscript, and approval of the article. All authors contributed to the article and approved the submitted version.

## Conflict of Interest

The authors declare that the research was conducted in the absence of any commercial or financial relationships that could be construed as a potential conflict of interest.

## Publisher's Note

All claims expressed in this article are solely those of the authors and do not necessarily represent those of their affiliated organizations, or those of the publisher, the editors and the reviewers. Any product that may be evaluated in this article, or claim that may be made by its manufacturer, is not guaranteed or endorsed by the publisher.
